# Preoperative Nutritional Conditioning of Crohn’s Patients—Systematic Review of Current Evidence and Practice

**DOI:** 10.3390/nu9060562

**Published:** 2017-06-01

**Authors:** Fabian Grass, Basile Pache, David Martin, Dieter Hahnloser, Nicolas Demartines, Martin Hübner

**Affiliations:** Department of Visceral Surgery, Lausanne University Hospital CHUV, 1011 Lausanne, Switzerland; fabian.grass@chuv.ch (F.G.); basile.pache@chuv.ch (B.P.); david.martin@chuv.ch (D.M.); dieter.hahnloser@chuv.ch (D.H.); demartines@chuv.ch (N.D.)

**Keywords:** Crohn’s, inflammatory bowel disease, nutrition, supplement, surgery, preoperative, complications

## Abstract

Crohn’s disease is an incurable and frequently progressive entity with major impact on affected patients. Up to half of patients require surgery in the first 10 years after diagnosis and over 75% of operated patients require at least one further surgery within lifetime. In order to minimize surgical risk, modifiable risk factors such as nutritional status need to be optimized. This systematic review on preoperative nutritional support in adult Crohn’s patients between 1997 and 2017 aimed to provide an overview on target populations, screening modalities, routes of administration, and expected benefits. Pertinent study characteristics (prospective vs. retrospective, sample size, control group, limitations) were defined a priori. Twenty-nine studies were retained, of which 14 original studies (9 retrospective, 4 prospective, and 1 randomized controlled trial) and 15 reviews. Study heterogeneity was high regarding nutritional regimens and outcome, and meta-analysis could not be performed. Most studies were conducted without matched control group and thus provide modest level of evidence. Consistently, malnutrition was found to be a major risk factor for postoperative complications, and both enteral and parenteral routes were efficient in decreasing postoperative morbidity. Current guidelines for nutrition in general surgery apply also to Crohn’s patients. The route of administration should be chosen according to disease presentation and patients’ condition. Further studies are needed to strengthen the evidence.

## 1. Introduction

Nutritional support strategies in malnourished patients became widely accepted tool to decrease postoperative morbidity in major gastrointestinal surgery [1,2]. Up to 85% of patients with Crohn’s disease awaiting surgery are malnourished as a consequence of active and disabling disease [3], impeding proper dietary intake and resorption [4,5,6]. Since up to 70% of Crohn’s patients at some point requires surgery [7,8], a bowel-sparing attitude is mandatory in order to prevent short bowel syndromes, malnutrition, and anemia [9,10]. Despite advances in non-surgical management of acute flares through multimodal concepts including biologics, antibiotics, and nutritional support, surgery remains a last treatment option for medically exhausted cases [11,12,13]. Interestingly, it should be emphasized that incidence of surgical procedures for Crohn’s disease did not decrease after introduction of infliximab [14]. Postoperative complication rates, including most feared intra-abdominal septic complications, reach 30% [15,16]. Nutritional guidelines for Crohn’s patients have been published by the European Society for Clinical nutrition and metabolism (ESPEN) in 2006 [17] and 2016 [4]. Most recommendations are based on consensus among experts or extrapolated from the general surgical population.

The aim of this present study was to systematically review scientific evidence over the last 20 years in an attempt to provide evidence-based recommendations.

## 2. Materials and Methods 

### 2.1. Data sources and Search Strategies

Main electronic databases including Medline (searched through PubMed), Embase, the Cochrane Database of Systematic Review and the Cochrane Central Register of Controlled trials were systematically searched. For searching PubMed, the medical subject heading (MeSH) were “(Crohn OR Crohn’s Disease OR inflammatory bowel disease) AND (nutrition OR conditioning OR nutritional support) AND (preoperative OR perioperative)”.

Electronic links were searched for related articles and cross-referencing of selected articles was performed by two authors (FG, BP). The trial registry http://clinicaltrials.gov was screened for relevant unpublished prospective trials. The search was limited to studies published between 1 January 1997 and 28 February 2017 for the following reasons: First, to provide evidence over the last 20 years, considering 10 years before the implementation of guidelines [17] and 10 years thereafter. Second, U.S. Food and Drug Administration approval of infliximab was granted in 1997 [14]. All kinds of original scientific reports were considered, and reviews and book chapters were also included. No language restrictions were applied for the original search, but studies providing only English abstracts were consequently excluded.

### 2.2. Study Selection (Inclusion and Exclusion Criteria)

Original studies and reviews reporting on pre- or perioperative nutritional support in Crohn’s disease were included. Excluded from the analysis were reports on general nutritional support in Crohn’s disease (including postoperatively) or in pediatric populations. The included manuscripts were divided in original scientific reports and reviews.

### 2.3. Data Extraction and Quality Assessment

Pertinent study characteristics (prospective vs. retrospective, sample size, control group, matching) were defined a priori and each manuscript was assessed for potential sources of bias. Two authors independently performed the literature search. The search terms were firstly identified in the title, and secondly in the abstract or medical subject heading. All studies of interest were obtained as full text articles and scrutinized thoroughly. Three authors made the final decision on inclusion of a study.

**Figure 1:**
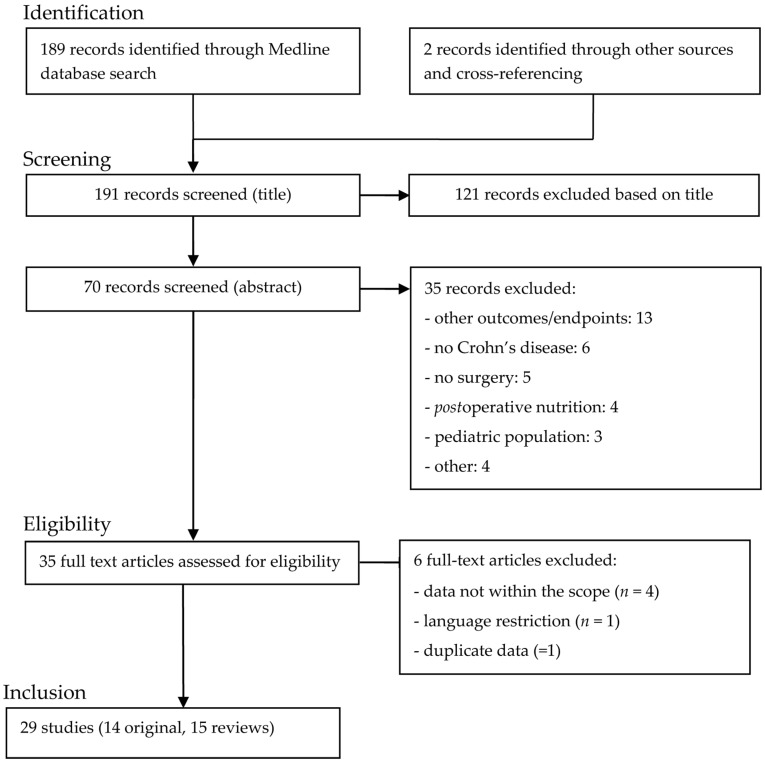
The selection process adhere to the guidelines outlined in the PRISMA statement [18].

Relevant data were extracted and documented in a database developed ad hoc for all publications. The following items were recorded for each study when available: authors, title, year of publication, disease presentation/surgical indication, details on nutritional regimen (type/formula/duration/timing), and potential limitations of original studies. Postoperative outcomes of interest were complications (overall, infectious/septic, non-infectious), recurrence rates, and changes in different nutritional parameters if available. Data are presented in accordance to the PRISMA statement [18] (Figure 1).

Based on the findings of this study, an algorithm was created for practical guidance.

### 2.4. Data Analysis

Meta-analysis of results was not feasible due to limited and heterogeneous original data. Instead, tables were created with descriptive statistics to display the most relevant findings of each original study and review to give a comprehensive overview of the most relevant results. 

## 3. Results

Electronic search of PubMed yielded 189 studies. By cross-referencing and through other data sources, two further studies [16,19] were identified matching the inclusion criteria. Of these 191 studies, 121 were excluded based on the title, and 35 further studies based on the abstract with reasons for exclusion displayed in Figure 1, remaining 35 full text articles assessed. Six were subsequently excluded for the following reasons: incomplete data or not within the scope of the present analysis (*n* = 4) [20,21,22,23], language restriction (*n* = 1) [24], and risk of double publication (*n* = 1) [25] (Figure 1). For final analysis, 14 original studies [26,27,28,29,30,31,32,33,34,35,36,37,38,39] and 15 reviews [4,5,17,19,40,41,42,43,44,45,46,47,48,49,50] were retained (Table 1, Table 2 and Table 3). All studies except one were indexed in PubMed. The one not indexed was a conference paper [51] from Cochrane Database with no detailed data thus was excluded for final analysis. Two studies were found on http://clinicaltrials.gov, one completed study from a Chinese group (NCT01540942) and one Canadian study which was not yet recruiting by 8 March 2017 (NCT02985489).

### 3.1. Methodological Assessment of Inlcuded Studies

#### 3.1.1. Study Design and Quality of Original Studies

Overall, 2141 patients with Crohn’s disease were reported in study and control groups, respectively (Table 1). Nine studies (64%) were retrospective [26,27,29,30,32,33,34,36,37] totaling 1783 patients. The remaining 358 patients were studied in 4 (29%) prospective [28,35,38,39] and 1 randomized controlled trial (RCT) [31]. In the only RCT, patients were not randomized to a nutritional regimen but to different endpoint measures, and thus no nutritional control group was available. Nutritional control groups were available in nine studies (64%) [26,28,29,30,32,33,35,37,38]. Only three studies (21%) [26,29,35] matched study and control groups, however, matching criteria were inconsistent among these studies. 

#### 3.1.2. Patients, Disease Presentation and Nutritional Details

In 12 studies (86%) nutritional support was administered preoperative only, ranging from two weeks up to three months. The remaining two studies [31,38] administered nutritional support pre- and postoperatively. Nutritional regimens were heterogeneous among included studies. Details on formulas are displayed in Table 2. In six studies (43%) [30,31,34,36,37,39], nutritional formulas were not or incompletely described. Six studies [26,28,29,32,33,39] evaluated the impact of exclusive enteral nutrition (EEN), four studies [34,35,37,38] reported on total parenteral nutrition (TPN) and four studies [27,30,31,36] combined different ways of nutritional support. Most studies (71%) [26,28,30,31,32,33,34,35,37,38] reported on severely sick and malnourished patients with either obstructing or fistulizing disease (Table 2). The remaining studies [27,29,36,39] optimized their cohorts or reported on low-risk patients.

### 3.2. Outcome

Five studies [26,29,32,33,35] showed significantly better results in terms of overall and infectious complications in groups undergoing preoperative nutritional therapy compared to control groups (Table 2). Among these studies, four [26,29,32,33] used EEN formulas and one [35] TPN. In the study of Heerasing et al. [26], 25% of patients could avoid surgery due to EEN induced remission and were bridged to alternative immunosuppressant therapy, but follow-up was limited to one year. Further effects of EEN were a significant decrease in CRP levels, surgical complications (8% vs. 32%) and infectious complications (abscess, collection, or leak, 3% vs. 20%) [4]. Wang et al. [29] showed an effect of EEN on different nutritional parameters (significant improvement of BMI, anemia and CRP levels), significantly lower infectious (21% vs. 44%) and non-infectious (26% vs. 51%) complication rates and less recurrence at six months (7% vs. 26%). Lower incidences of total (19% vs. 29%) and specific infectious complications (wound infection, abscess, and leak) were observed in the study of Li et al. [32] when comparing the steroid-weaned EEN group with the steroid-weaned control group. Further, supplemented patients needed less emergency surgeries compared to the different control groups. Li et al. [33] demonstrated a significant improvement of albumin and CRP levels after EEN therapy and at three months postoperatively, intra-abdominal septic complications were significantly lower (4% vs. 18%). In the cohort of Jacobson [35], patients pre-treated with TPN showed clinical remission and improved nutritional status (albumin, weight) at the time of surgery and no serious early (30 days) postoperative complications were observed in these 15 consecutive patients, contrarily to 28% in the matched control group.

Three studies [28,30,37] could not demonstrate differences in outcome due to unequal nutritional baseline conditions between nutritional and control groups, as detailed in Table 2. Three studies [31,36,39] without control groups compared their results to historical controls of other authors and found overall complication rates of 14% [39] and 18% [31]. However, these two studies were designed to compare nutritional endpoints [31] and anastomotic techniques [39] rather than focusing on impact of nutritional support. The preoperatively optimized cohort of Zerbib et al. [36] with 64% of patients receiving nutritional support (in combination with bowel rest, weaning of steroids, abscess drainage, and antibiotics) presented an overall morbidity of 18% and a low rate of fecal diversion. Another study without a control group by Guo et al. [27] identified EEN of <3 months, preoperative anemia and bacteria in fistula tract as independent risk factors for surgical site infection (31%), while preoperative abscess drainage represented a protective factor. Another study did not compare nutritional regimens [34], but reported TPN in combination with antibiotics, drainage, and postponed surgery in patients with penetrating disease which led to similar complication rates compared to patients with non-perforating disease (13% vs. 11%). The cohort of Yao et al. [38] was severely malnourished and half of patients were supplement by TPN one week before surgery and continued two weeks postoperatively. IgM levels decreased and BMI increased significantly in the study group, while no changes were observed in the control group. No difference was found regarding overall postoperative complications between the two groups (27% each), but a six month follow-up showed that the rate of resuming work was higher in the study group [38].

### 3.3. Reviews

Fifteen reviews, guidelines or book chapters were retained [4,5,17,19,40,41,42,43,44,45,46,47,48,49,50] (Table 3). Most of them (80%) [5,19,40,41,42,43,44,46,47,48,49,50] were narrative, and only two (13%) [4,17] did perform systematic search to provide official guidelines by the ESPEN society. The more recent recommendations advocate:

- enteral nutrition always preferred over parenteral nutrition in malnourished patients (weight loss >10–15% within six months, BMI <18.5 kg/m^2^, albumin <30 g/L)
- postpone surgery for 7–14 days if possible
- parenteral nutrition should be used as supplementary to enteral nutrition if >60% energy needs cannot be met via the enteral route

These recommendations are congruent and 1:1 extrapolated from guidelines on enteral nutrition [53,54]. 

All reviews are consistent among each other regarding conclusions and agree on the importance of perioperative nutritional support. Further, they provide recommendations in line with current guidelines: if compared, enteral nutrition should be the preferred route of administration. All reviews underline the importance of a multimodal approach (preoperative optimization). Evidence-based recommendations however are scarce, since no solid evidence is available, and all authors agree that more high quality studies are needed to establish solid recommendations. Further, the impact of specific components of nutritional supplements should be studied to provide further evidence-based formulas [5,41]. 

## 4. Discussion

This systematic review scrutinized available evidence over the last 20 years to provide evidence-based guidelines for perioperative nutritional support in patients suffering from Crohn’s disease. Fourteen original studies evaluated nutritional support in mostly severely ill patients, and a large heterogeneity was observed among studies regarding type, formula, and timing of nutrition. Only few prospective studies were available, and a randomized controlled study comparing different nutritional strategies was not to date. Hence, comparison between studies is delicate, and conclusions should be drawn cautiously. Even though nutritional support strategies were different, all studies presented encouraging results and emphasized the importance of nutritional support within a multimodal preoperative optimization concept. Some general principles in patients suffering from Crohn’s disease must be discussed, including particularities of Crohn’s patients, screening modalities, and current guidelines, which are discussed and compared to the evidence provided by this systematic review.

### 4.1. Particularities in Surgery for Crohn’s Disease

Patients suffering from Crohn’s disease are a particular subset of patients in many ways. At the time of surgery, most patients are treated by immunomodulating drugs, present with intra-abdominal infections, and are anemic and malnourished [55]. In a recent meta-analysis, steroid use, low albumin level, preoperative surgical history, and preoperative abscess were retained as risk factors for adverse surgical outcome [56]. Besides steroids and thiopurines, biologics such as anti-TNF provide new treatment options for disease control [57]. However, the influence of these drugs on postoperative outcome is matter of debate. While Fumery et al. described an increased risk of complications [15], a recent meta-analysis did not find any association between immunomodulating therapy and postoperative outcome [58]. Malnutrition on the other hand is common (up to 85%) among Crohn’s patients awaiting surgery and is a well-known risk factor for adverse postoperative outcome in surgical patients in general [59]. For Crohn’s patients needing surgery, anastomotic dehiscence, intraabdominal abscess, and fistula, regrouped as intraabdominal septic complications, represent most feared complications [60,61]. Intraabdominal septic complications hinder the postoperative course in up to 20% of patients with potentially severe consequences [15,62] and either reoperation or percutaneous drainage is needed in most cases. Hence, efforts to improve modifiable risk factors before surgery are of utmost importance.

### 4.2. Guidelines for Perioperative Nutrition and Preoperative Optimization

By the time of official ESPEN guidelines publication in 2006 [17], specific data on the effect of perioperative nutrition was lacking. Considerable evidence on nutritional support in general gastrointestinal surgery and in critically ill patients led by extrapolation to the recommendation to treat Crohn’s patients accordingly [17]. This message was reinforced 10 years later by revised guidelines [4]. Hence, the ESPEN guidelines on enteral nutrition for surgery [53], published in 2006 and 2017 [54], do apply for Crohn’s patients if they tolerate nutritional supplements to meet their metabolic needs. Most recommendations concerning enteral nutrition were elaborated on firm evidence and are hence highly recommended [53,54]. Whenever possible, the route of administration should be enteral, which is also advocated for patients with Crohn’s disease [17]. This was previously emphasized by a review on nutritional support strategies in Crohn’s disease [63]. Guidelines for parenteral nutrition [64] in the perioperative phase do likewise apply for Crohn’s patients if metabolic needs are not met by enteral nutrition alone or if disease presentation at the time of scheduled surgery impedes enteral nutrition (e.g., intestinal obstruction or high output fistula). Nutritional guidelines merge with enhanced recovery after surgery (ERAS) guidelines [65], which are beneficial for surgical patients regarding outcome, length of stay, and costs. Recent reports suggested that enhanced recovery combined with minimally invasive techniques may lead to further improvements in surgical outcomes of Crohn’s patients [66,67]. Whenever possible, elective surgical patients should be treated according to the ERAS protocol: avoidance of long term fasting, integration of nutritional strategies into the overall management of the patient, metabolic homeostasis, and early mobilization [4].

Nutritional strategies need to be part of a concept called preoperative optimization, including weaning of steroids if possible, drainage of percutaneous abscesses if applicable, and intravenous antibiotics if indicated [68]. Several of the studies retained for the present analysis presented promising data within such a multimodal approach [27,36,39,40,42]. Thus, surgery has to be delayed if possible in order to ensure best conditions. In case of emergency, EN or PN should start postoperatively [4].

### 4.3. Nutritional Screening

Several original studies [30,37,38] retained for the present analysis reported on nutritional screening tools or markers to provide nutritional support, especially by emphasizing the importance of body mass index (BMI). They all identified BMI as a follow-up tool of nutritional status during parenteral nutritional therapy. Recent guidelines advocate BMI <18.5 kg/m^2^, weight loss >10–15% within six months and serum albumin <30 g/L as best reflectors of severe undernutrition in Crohn’s disease [4]. Concerning screening, ESPEN guidelines for Inflammatory Bowel Disease [4] recommend that Crohn’s patients should be screened for malnutrition as patients undergoing general surgery [53,54] through validated screening tools. Particularly recommended are the Nutritional Risk Score (NRS) [69] and the Malnutrition Universal Screening Tool (MUST) [70]. Patients with a NRS ≥3 are considered to be at risk for gastrointestinal surgery [71].

### 4.4. Further Nutritional Strategies for Perioperative Support in Crohn’s Patients

Two concepts need special consideration as result of this systematic review: EEN and TPN. Interestingly, most included studies reported on either EEN or TPN or a combination of both. EEN, either in elemental or polymeric form, has a direct anti-inflammatory effect [72], promotes mucosal healing [73], modifies intestinal microflora [74], and might decrease the antigenic load through bowel rest. EEN can induce clinical remission in pediatric and adult patients [75,76], as observed by Heerasing et al. [26] in 25% of patients awaiting surgery. In a recent review analyzing EEN in non-surgical Crohn’s patients [77], EEN has been associated with remission rates of up to 80%. Wang et al. [29] observed decreased recurrence rates at six months in the EEN group, however, clinical recurrence was similar two years after surgery in both groups. Li et al. [32] and Smedh et al. [39] further presented interesting data on EEN allowing subsequent steroid-weaning, contributing to lower complication rates in EEN-groups in these studies. Disease presentations were severe in all studies with EEN [26,28,32,33,39] but one, [29], and might thus be particularly useful in this context (Table 2).

TPN was mainly used for penetrating disease in the study of Bellolio et al. [34], and Yao et al. [38] and Jacobson [35] treated patients with obstructing disease to observe improved immunity and clinical remission. Concerning formulas and timing, data was heterogeneous. Hence, no solid conclusions can be drawn. As a consequence, guidelines on parenteral nutrition [64] should be used for guidance. Schwartz [41] emphasized the need for larger prospective trials to strengthen the evidence. With this respect and due to lacking data, parenteral nutrition should be reserved for patients who are unable to cover their energetic needs by enteral nutrition.

Further considerations regarding routes of administration and associated potential complications have been published before [63].

### 4.5. Particularities in Perioperative Nutrition for Crohn’s Disease

**Figure 2:**
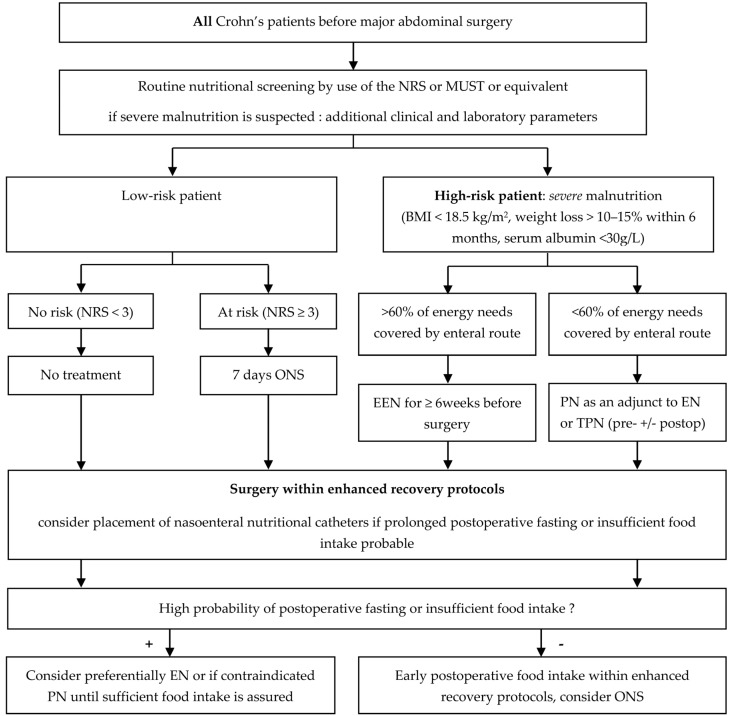
Nutritional treatment algorithm for preoperative nutritional screening and perioperative nutrition in digestive surgery in Crohn’s patients. Abbreviations: NRS—Nutritional Risk Score, MUST—Malnutrition Universal Screening Tool; EEN—exclusive enteral nutrition; PN—parenteral nutrition; EN—enteral nutrition; TPN—total parenteral nutrition; preop—preoperative; postop—postoperative.

Despite the particularities of Crohn’s disease and potential clinical discrepancies with the general surgical population including disease flares at time of surgery, exhaustive immunomodulating and medical treatment, and unfavorable baseline conditions, guidelines on enteral and parenteral nutrition including screening modalities, nutritional support strategies, and nutritional follow-up can be extrapolated to Crohn’s patients. However, severe malnutrition in high-risk patients or inability to cover energy needs in patients with obstructing or fistulizing disease might impede conventional nutritional support (including oral nutritional supplements and immunonutrition) [4]. In these circumstances, specific nutritional support strategies including EEN or TPN have to be discussed. The following algorithm gives an overview on treatment suggestions considering available guidelines and the evidence of this systematic review (Figure 2).

Several limitations of the present study need to be mentioned. Due to heterogeneity of data and modest study quality of original studies regarding nutritional treatment strategies, solid conclusions cannot be drawn, and further high-quality evidence will be needed. The suggested treatment algorithm (Figure 2) should thus rather help in decision-making than provide formal recommendations. 

## 5. Conclusions

Perioperative nutrition in Crohn’s patients awaiting surgery should be considered as a mandatory adjunct within preoperative optimization strategies. Guidelines including enteral nutrition and perioperative care for general surgery do also apply to Crohn’s patients. Encouraging data for exclusive enteral or total parenteral nutrition, especially regarding induction of surgery-preventing disease remission and decreased recurrence, call for further high-quality studies.

## Figures and Tables

**Table 1:** Original studies on preoperative nutritional support in Crohn’s disease patients.

Author	Year	Design	Control	Matching	*N*	Limitations
Heerasing [26]	2017	Retrospective	Yes	Yes	114	Incomplete matching for disease severity
Guo [27]	2017	Retrospective	No	na	118	No outcome data other than SSI
Beaupel [28]	2017	Prospective	Yes	No	56	Comparison high-risk to low-risk patients
Wang [29]	2016	Retrospective	Yes	Yes	81	Potential selection bias for study group
Zhang [30]	2015	Retrospective	Yes	No	64	Comparison high-risk to low-risk patients
Zhu [31]	2015	RCT	No	na	108	No nutritional control group
Li [32]	2015	Retrospective	Yes	No	708	Potential selection bias, <10% laparoscopy
Li [33]	2014	Retrospective	Yes	No	123	No dietary information of control group
Bellolio [34]	2013	Retrospective	No	No	434	No nutritional control group
Jacobson [35]	2012	Prospective	Yes	Yes	120	No matching for disease severity
Zerbib [36]	2010	Retrospective	No	Na	78	Heterogeneous study groups
Grivceva [37]	2008	Retrospective	Yes	No	63	Composition of diets not specified
Yao [38]	2005	Prospective	Yes	No	32	Small sample size
Smedh [39]	2002	Prospective	No	na	42	Small sample size, no nutritional control group

Abbreviations: RCT—randomized controlled trial, SSI—surgical site infection, *N*—number of included patients, na—not available

**Table 2:** Nutritional details and outcome of original studies.

Author	Disease	Type/Formula	Timing	Duration	Groups/Cohort	Main results (Nutritional Group)
Heerasing [26]	P/F	EEN ^1^	Pre	6 w (mean)	EEN pre-treatment group vs. straight to surgery group	Nine-fold decreased infectious complications, shorter operating time
Guo [27]	F	PN+EN ^2^	Pre	3 m	Preop optimized cohort (nutritional support, steroid weaning, abscess drainage, antibiotics)	EEN <3 m retained as independent risk factor for SSI
Beaupel [28]	P/F	ANS-TGF-b2 (EEN)	Pre	3 w (median)	Supplemented high-risk (steroids, malnutrition) vs. non supplemented low-risk patients	Similar overall and infectious complications
Wang [29]	FS	EEN ^2^	Pre	4 w	Low-risk patients (no immunosuppression, no inflammation) in both groups (EEN vs. non-EEN)	Decreased overall and infectious complications, less recurrence at 6 m
Zhang [30]	F/O	TPN or PN or EN (na)	Pre	3 w (median)	Fortified nutrition support group (lower BMI, higher CDAI) vs. non-supplemented control group	Similar postoperative septic complications (3 m)
Zhu [31]	F/P	EEN^2^ +/-PN +/-TPN (na)	Pre Post	4 w 4 w	Supplementation in all patients, randomization and blinding for two endpoints: ROI and IOM	Similar complications (4 w) in ROI group = better endpoint than IOM, less complications than historical controls
Li [32]	R/F/O/P	EEN ^2^	Pre	4 w	Immunosuppressants-treated EEN patients vs. different non-supplemented control groups	Decreased overall and infectious complications (30 days) in EEN-group
Li [33]	F	EEN ^3^	Pre	3 m	EEN group vs. normal diet group, abscess-drainage in all patients	Decrease of intra-abdominal septic complications at 3 m
Bellolio [34]	P/N-P	TPN (na)	Pre	na	TPN for bowel rest in patients with penetrating disease vs. few TPN in non-penetrating disease	Similar complication rates in both groups, beneficial effect of TPN and bowel rest
Jacobson [35]	O	TPN [52]	Pre	46 days (mean)	Matched cohort of preoperative TPN vs. straight to surgery group	Clinical remission achieved, postoperative complications (30 days), decreased
Zerbib [36]	F/P	EN ^4^/TPN (na)	Pre	2 w/3 w	Preop optimized cohort (nutritional support, steroid weaning, abscess drainage, antibiotics)	Low postoperative morbidity (30 days) and stoma rate within a standardized pathway
Grivceva [37]	FS	TPN (na)	Pre	12 days (mean)	PN group (with lower BMI and higher CDAI) vs. non-supplemented control group	Improvement of BMI/CDAI, no difference in outcome
Yao [38]	O	TPN ^5^	Peri	3 w	Severely malnourished cohort (BMI <15), TPN group vs. non-supplemented control group	TPN ameliorates immunity, reverses malnutrition (BMI), facilitates recovery
Smedh [39]	F/FS	EEN (na)	Pre	3–6 w	Preoperative optimized cohort (EEN in 50% of patients, steroid weaning, abscess drainage)	Few postop complications (30 days) compared to historical control groups

^1^ Modulen IBD (Nestle, Vevey, Switzerland), ^2^ Peptisorb Liquid, Enteral Nutrition Suspension; Nutricia Company, Amsterdam, the Netherlands, ^3^ Peptison Liquid, Nutricia Company, (Shanghai, China), ^4^ elemental diet >30 kcal/kg ideal body weight/day, ^5^ nitrogen 0.2 g/kg/day, 30 kcal/kg/day, fat 40%; glucose 60%. Abbreviations: P—Penetrating, F—Fistulizing, FS—Fibrous Stenosis, O—Obstructing, R—Refractory Disease, EEN—Exclusive Enteral Nutrition, PN—Parenteral Nutrition, EN—Enteral Nutrition, TPN —Total Parenteral Nutrition, na—not available, w—weeks, m—months, d—days, Preop—Preoperative, Postop—Postoperative, BMI—Body Mass Index, CDAI—Crohn’s Disease Activity Index, ROI—reduction of inflammation, IOM—improvement of malnutrition.

**Table 3:** Reviews on preoperative nutritional support in Crohn’s disease patients.

Author	Year	Design	Aim/Conclusions
Forbes [4]	2016	Guidelines	64 recommendations to guide nutritional support in IBD patients.
Nguyen [5]	2016	N. Review	Preoperative optimization by enteral and parenteral nutrition mandatory. Timing, route of administration, type, duration debated.
Nickerson [40]	2016	N. Review	Perioperative optimization imperative for favorable postoperative outcome.
Schwartz [41]	2016	N. Review	Evidence in favour of PN, but larger trials needed.
Montgomery [42]	2015	N. Review	Recommendations for nutritional assessment and preoperative optimization.
Horisberger [43]	2015	Book chapter	Preoperative protein supplements (at least one week) beneficial.
Crowell [44]	2015	N. Review	Preoperative optimization (nutritional support, abscess drainage) prevent septic complications and early recurrence.
Spinelli [45]	2014	N. Review	Preoperative optimization crucial for surgical outcome, preoperative enteral nutrition for at least 10–14 days to prefer over TPN.
Triantafillidis [19]	2014	N. Review	Indications for TPN are the same as in every major surgical patient.
Sharma [46]	2013	N. Review	Enteral support (immunonutrition and elemental diet) preferred over TPN.
Iesalnieks [47]	2012	N. Review	Preoperative enteral nutrition might be beneficial, more evidence needed.
Wagner [48]	2011	N. Review	EN preferred, preoperative and postoperative PN remain alternatives. Consider immunonutrition, fish oils, and probiotics.
Efron [49]	2007	N. Review	Perioperative TPN might be beneficial, more high quality studies needed.
Lochs [17]	2006	Guidelines	No specifics for Crohn’s patients, perioperative nutrition as in general GI surgery.
Husain [50]	1998	N. Review	Nutrition has a critical benefit in postoperative Crohn’s disease.

Abbreviations: N—Narrative, Postop—postoperative, PN—Parenteral Nutrition, TPN—Total Parenteral Nutrition, EN—Enteral Nutrition, IBD—Inflammatory Bowel Disease, GI—gastrointestinal.
